# Periodontal pathogens and obesity in the context of cardiovascular risks across age groups

**DOI:** 10.3389/froh.2024.1488833

**Published:** 2025-01-09

**Authors:** Georgy Leonov, Yurgita Varaeva, Elena Livantsova, Andrey Vasilyev, Olga Vladimirskaya, Tatyana Korotkova, Dmitry Nikityuk, Antonina Starodubova

**Affiliations:** ^1^ Department of Cardiovascular Pathology and Diet Therapy, Federal Research Centre for Nutrition, Biotechnology and Food Safety, Moscow, Russia; ^2^ Department of Microbiology, Central Research Institute of Dental and Maxillofacial Surgery, Moscow, Russia; ^3^ Institute of Dentistry, I.M. Sechenov First Moscow State Medical University, Moscow, Russia; ^4^ Therapy Faculty, Pirogov Russian National Research Medical University, Moscow, Russia

**Keywords:** obesity, cardiovascular disease, periodontal pathogens, *P. gingivalis*, biomarkers

## Abstract

**Background:**

Cardiovascular diseases (CVDs) are the leading cause of mortality and morbidity among noncommunicable diseases. Over the past decade, there has been a notable increase in the prevalence of CVDs among young individuals. Obesity, a well-known risk factor for CVDs, is also associated with various comorbidities that may contribute to cardiovascular risk. The relationship between periodontal pathogens and CVD risk factors, including obesity, smoking, lipid metabolism disorders, and inflammatory markers, remains underexplored.

**Methods:**

This study examined the relationship between six periodontal pathogens (*Porphyromonas gingivalis, Aggregatibacter actinomycetemcomitans, Treponema denticola, Tannerella forsythia, Prevotella intermedia, and Fusobacterium nucleatum*) and CVD risk factors among 189 subjects stratified by age and body mass index (BMI). Body composition was assessed via bioimpedance analysis, and blood samples were analyzed for lipid profiles, glucose, and proinflammatory cytokines. Oral samples were collected for polymerase chain reaction (PCR) analysis to identify periodontal pathogens. Cardiovascular and diabetes risk scores were calculated using the SCORE and FINDRISC scales.

**Results:**

The prevalence of periodontal pathogens in the population was 33.0% for *P. gingivalis*, 47.8% for *P. intermedia*, 63.4% for *A. actinomycetemcomitans*, 46.6% for *T. forsythia*, 46.6% for *T. denticola*, and 89.2% for *F. nucleatum*. Significant age- and BMI-related differences were observed in pathogen prevalence, particularly with *P. gingivalis*, *P. intermedia*, and *T. denticola*. Young obese individuals exhibited a higher prevalence of *P. intermedia* and *T. forsythia*. *P. gingivalis* was found to be associated with hypertension and dyslipidemia, while *P. intermedia* was linked to hypertension and obesity. *T. denticola* was associated with obesity, dyslipidemia and smoking, whereas *T. forsythia* was linked to dyslipidemia alone.

**Conclusions:**

This study highlights the potential connection between periodontal pathogens and risk factors associated with cardiovascular disease, including smoking, elevated BMI, increased adipose tissue, hypertension, and dyslipidemia. Further research is required to determine the causal relationships between oral microbiome dysbiosis, obesity and, systemic diseases and to develop an effective strategy for preventing oral health-related CVD risk factors in young adults.

## 1 Introduction

Cardiovascular diseases (CVDs) are the leading cause of mortality and morbidity among noncommunicable diseases, representing a significant global health challenge (1). In recent decades, the prevalence of CVDs has notably increased among individuals under 55 years of age, with a marked rise in cases of myocardial infarction and stroke within this demographic (2). A substantial body of evidence indicates that cumulative exposure to CVDs risk factors from childhood through young adulthood significantly contributes to this trend (3).

Obesity is a trigger for many diseases, such as non-alcoholic fatty liver disease, non-alcoholic steatohepatitis, cardiovascular diseases, diabetes, and some types of cancer (4, 5). The relationship between adipose tissue and CVDs is mediated through both direct and indirect pathways associated with obesity-related comorbidities. For instance, obesity is a well-established risk factor for several traditional CVDs, such as atherogenic dyslipidemia, hypertension, and diabetes (6). Additionally, obesity-related obstructive sleep apnea can elevate the risk of CVDs through mechanisms involving hypoxia, cardiac arrhythmias, insulin resistance, and hypertension (7). There is some evidence of a connection between oral microorganisms, obesity and metabolic disorders, both at the level of overall diversity and individual species (8, 9).

The physiology and ecology of the microbiota are intimately linked to those of the host at both the macro and microscopic levels (10). The human oral microbiome, comprising bacteria, archaea, viruses, fungi, and protozoa, includes over 700 identified species of microorganisms (11). Oral bacteria primarily exist as structured communities of aggregated bacterial cells (biofilms) (12). Dysbiosis of the oral microbiota represents a complex, multifactorial displacement of native microorganisms within the oral cavity, where potentially pathogenic species supersede commensal flora (13–15). Opportunistic anaerobic bacteria involved in periodontal diseases (PDs) exert significant negative effects on systemic health. PDs are microbial-induced inflammatory and multifactorial chronic immunological diseases leading to damage to the gums, periodontal ligaments, and alveolar bone (16). At present, a multitude of periodontopathic organisms have been identified, with ongoing research elucidating their characteristics and pathogenic potential (17, 18). The most substantial evidence for negative impacts on systemic disorders, particularly cardiometabolic health, was observed in studies involving *Porphyromonas gingivalis*, *Aggregatibacter actinomycetemcomitans*, *Treponema denticola*, *Tannerella forsythia*, *Prevotella intermedia*, and *Fusobacterium nucleatum* (19–21).

There are several potential reasons for the association of CVDs and periodontal diseases: systemic inflammation, the direct damaging effect of microorganisms and their metabolites entering the bloodstream, as well as alterations in the intestinal microbiome due to the transfer of oral bacteria (22). Systemic inflammation is a potential underlying mechanism of the association between oral diseases and increased risk of cardiovascular disease (23, 24). Elevated inflammatory markers, such as erythrocyte sedimentation rate (ESR), C-reactive protein (CRP), and interleukin-6 (IL-6), have been correlated with higher cardiovascular morbidity and mortality (25). According to some data, periodontitis-associated systemic inflammation can cause vascular dysfunction (26). The hematogenous route facilitates the spread of oral bacteria to distant organs, as the ulcerated epithelium of the periodontal pocket allows microorganisms and their toxins to enter the systemic circulation, leading to bacteremia (27). Recent research has shown that *P. gingivalis, P. intermedia, A. actinomycetemcomitans, T. forsythensis, and T. denticola* and others are present in 20%–70% of carotid atheromas (28). Cross-sectional studies have demonstrated a higher incidence of atherosclerotic complications in patients with periodontal disease. In the NHANES III cohort, severe periodontal disease was associated with an almost 4-fold higher incidence of myocardial infarction than in patients without periodontal disease (29). Moreover, a study involving 52,677 hypertensive participants indicated that dental caries is linked to an elevated CVD (30). Oral microorganisms may serve as a new biomarker for CVD and metabolic disorders. Interdisciplinary collaboration can improve the early diagnosis and treatment of dental and systemic diseases, including CVDs.

The objective of this study was to examine the prevalence of periodontal pathogens, specifically *P. gingivalis*, *P. intermedia*, *A. actinomycetemcomitans*, *T. forsythia*, *T. denticola*, and *F. nucleatum*, in age- and obesity-specific groups. Additionally, the study aimed to investigate the correlation between the presence of these bacteria and risk factors associated with cardiovascular disease. These risk factors include obesity, smoking, lipid disorders, and proinflammatory cytokines. We also calculated cardiovascular risk (relative risk of cardiovascular disease and SCORE), and the Finnish Diabetes Risk Index (FINDRISC).

## 2 Materials and methods

### 2.1 Ethical aspects

The study was conducted in accordance with the Declaration of Helsinki, and approved by the Local Ethics Committee of the Federal Research Center of Nutrition, Biotechnology and Food Safety (protocol code N3/2020 dated on 02/10/2020). The study period was from March 2020 to November 2023. Informed consent was obtained from all subjects involved in the study. The collected samples were analyzed in a de-identified manner in order to ensure the confidentiality of the participants.

### 2.2 Subjects and study design

**Figure 1:**
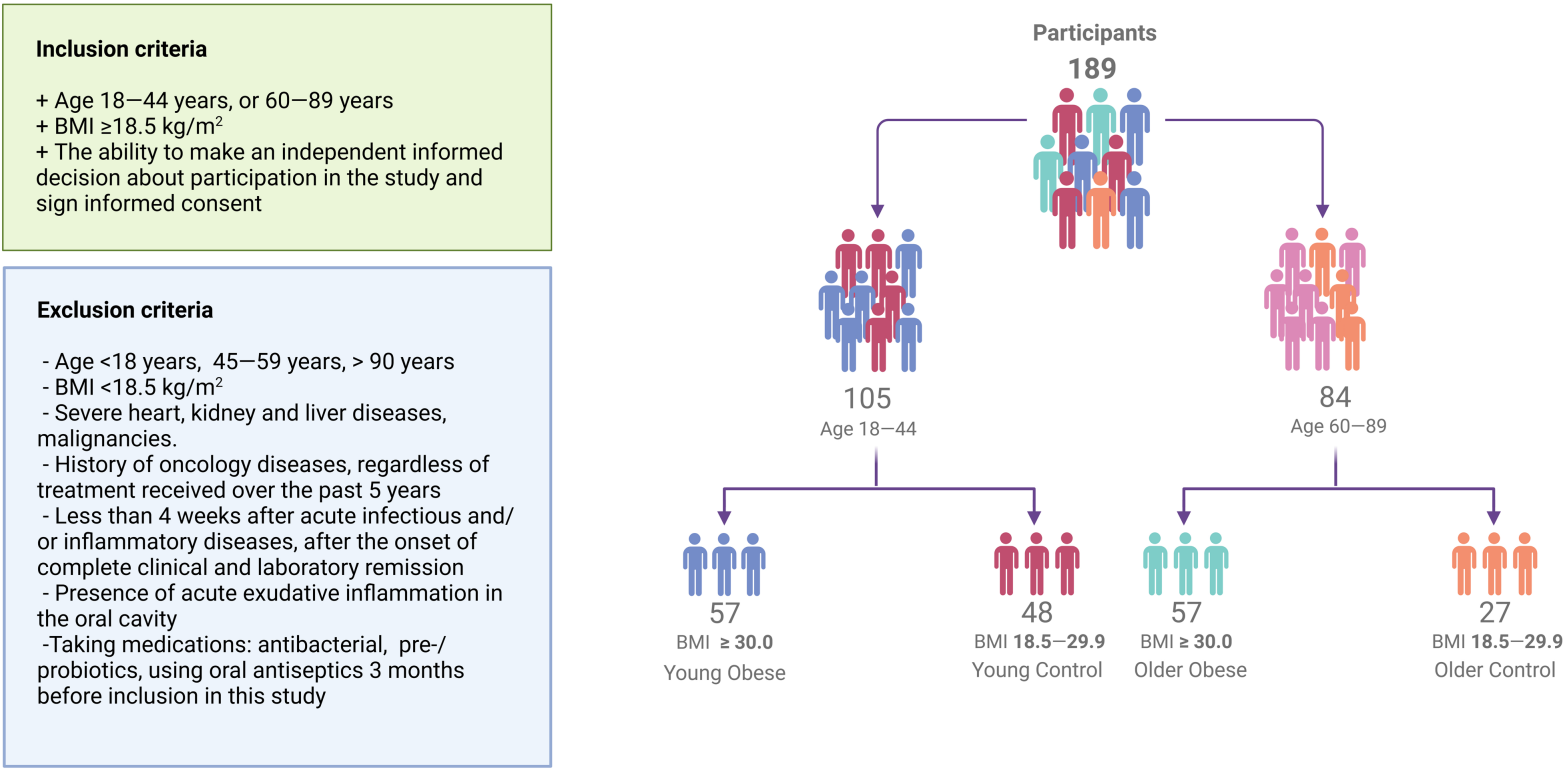
Flowchart visualizing participant recruitment. A total of 189 participants were enrolled in the study. Individuals were stratified into age and weight-adjusted groups.

The study included 189 Caucasian subjects [44 men (23%), mean age 48 ± 21 years, mean BMI 30.1 ± 7.7 kg/m^2^] stratified into groups based on age and body mass index (BMI) (31, 32). Due to the lack of published data, the preliminary sample size calculation was uninformative. As a result, as much as possible eligible participants were enrolled. 105 individuals were young (18–45 years), of whom 57 were obese (BMI≥30 kg/m^2^; Young Obese) and 48 were not obese (BMI 18.5–29.9 kg/m^2^; Young Control). 84 participants were older (60–84 years), of whom 57 were obese (BMI≥30 kg/m^2^; Older Obese) while the remaining 27 were non-obese (BMI 18.5–29.9 kg/m^2^; Older Control). Subjects aged 45–60 years were not included in the study to make the differences between groups more prominent and to exclude overlap between groups. All participants underwent examination at the Nutrition Clinic of the Federal Research Centre for Nutrition, Biotechnology and Food Safety. The self-reported oral health data and dental care usage information were obtained through the completion of an electronic questionnaire, in which the participants were required to select the most appropriate answer option. The questionnaire included items on the presence of bruxism, bleeding on brushing, dentin hypersensitivity, use of dentures, and frequency of dental visits. Cardiovascular risk was calculated using the Systematic Coronary Risk Evaluation (SCORE) risk scales. The diabetes risk score of each individual was calculated by the Finnish Diabetes Risk Score (FINDRISC tool) (33). The flowchart illustrating the methodology for participant allocation, as well as the inclusion and exclusion criteria, is presented in Figure 1.

### 2.3 Body composition measurements

Body weight and height were measured on a medical scale and stadiometer and performed as kg and m. BMI was calculated from weight and height using the BMI = weight (kg)/Height^2^ (m^2^) formula. Body fat mass (kg), muscle mass (kg), relative fat mass (%) etc. were measured by bioimpedance analysis on InBody 770 analyzer (Inbody Co. Ltd, Republic of Korea). The patient is required to fasten for at least 4 h in advance. In addition to the directly measured parameters, the fat-to-muscle ratio was calculated (34).

### 2.4 Glucose, lipid profile, and cytokines determinations

Venous blood was drawn by qualified medical personnel from each of the participants after overnight fasting from the antecubital vein using Vacutainer tubes (Unimed, Russia) for biochemical and enzyme-linked immunosorbent assay (ELISA) analysis of the serum. The results of the lipid profile (Triglycerides — TG, HDL-c, LDL-c, VLDL-c, and total cholesterol — TC), glucose, aspartate aminotransferase (AST), alanine aminotransferase (ALT), uric acid were provided by standard laboratory procedures on «KONELAB Prime 60i» Laboratory analyser (Thermo Fisher Scientific, USA). In addition to the directly measured parameters, we calculated the following additional indices: non-HDL, LDL/HDL ratio, TG/HDL ratio, TG/LDL ratio, and the atherogenic index (35–37). Serum levels of Interleukin-1 beta (IL-1β), Interleukin 6 (IL-6), Tumor necrosis factor alpha (TNF-α) were detected using ELISA kits (Cloude-Clone Corp., China) following the manufacturer's instructions.

### 2.5 Oral samples collection

Oral samples were collected in the morning, at least 8 h after the last tooth brushing and food/liquid intake. Participants were asked to rinse their mouths with clean and sterile water and waited for approximately 5 min. Biofilm samples were collected from the outer surface of teeth and supragingival plaque for 30 s using sterile cotton swabs. Cotton swabs were placed in one tube containing 1.5 ml of phosphate-buffered saline (PBS) and mixed for 30 s. Saliva samples were collected in sterile polypropylene tubes using the spitting method (3–5 ml over 3 min) (38). Biofilm and saliva samples were pooled and stored at −80°C until nucleic acid extraction was performed.

### 2.6 PCR analysis

DNA extraction was performed by the phenol-chloroform method using the Lira + kit (Biolambix, Russia) according to the manufacturer's instructions. A Nanodrop 1,000 spectrophotometer (Thermo Fisher Scientific, Waltham, MA, USA) was used to assess both the purity of DNA (via absorption ratios of the extracts at A260/A280) and the quantity of DNA. Then, using the specific 16S rRNA primers described in Table 1, the analysis of microbiota (P*. gingivalis, P. intermedia, A. actinomycetemcomitans, T. forsythia, T. denticola, and F. nucleatum*) was examined by PCR (39).

**Table 1:** Oligonucleotides used for PCR analysis.

Bacterial species	Primers	Annealing temperature, °C	Product length, bp
*P. gingivalis*	for- *AGGCAGCTTGCCATACTGCG*	65	404
	rev- *ACTGTTAGCAACTACCGATGT*		
*P. intermedia*	for- *CGTGGACCAAAGATTCATCGGTGGA*	64	259
	rev- *CCGCTTTACTCCCCAACAAA*		
*A.actinomycetemcomitans*	for- *GCTAATACCGCGTAGAGTCGG*	68	443
	rev-*ATTTCACACCTCACTTAAAGGT*		
*T. forsythia*	for- *GCGTATGTAACCTGCCCGCA*	60	641
	rev- *TGCTTCAGTGTCAGTTATACCT*		
*T. denticola*	for- *TAATACCGAATGTGCTCATTTACAT*	60	316
	rev- *TCAAAGAAGCATTCCCTCTTCTTCTTA*		
*F. nucleatum*	for- *AGAGTTTGATCCTGGCTCAG*	60	360
	rev- *GTCATCGTGCACACAGAATTGCTG*		

**Figure 2:**
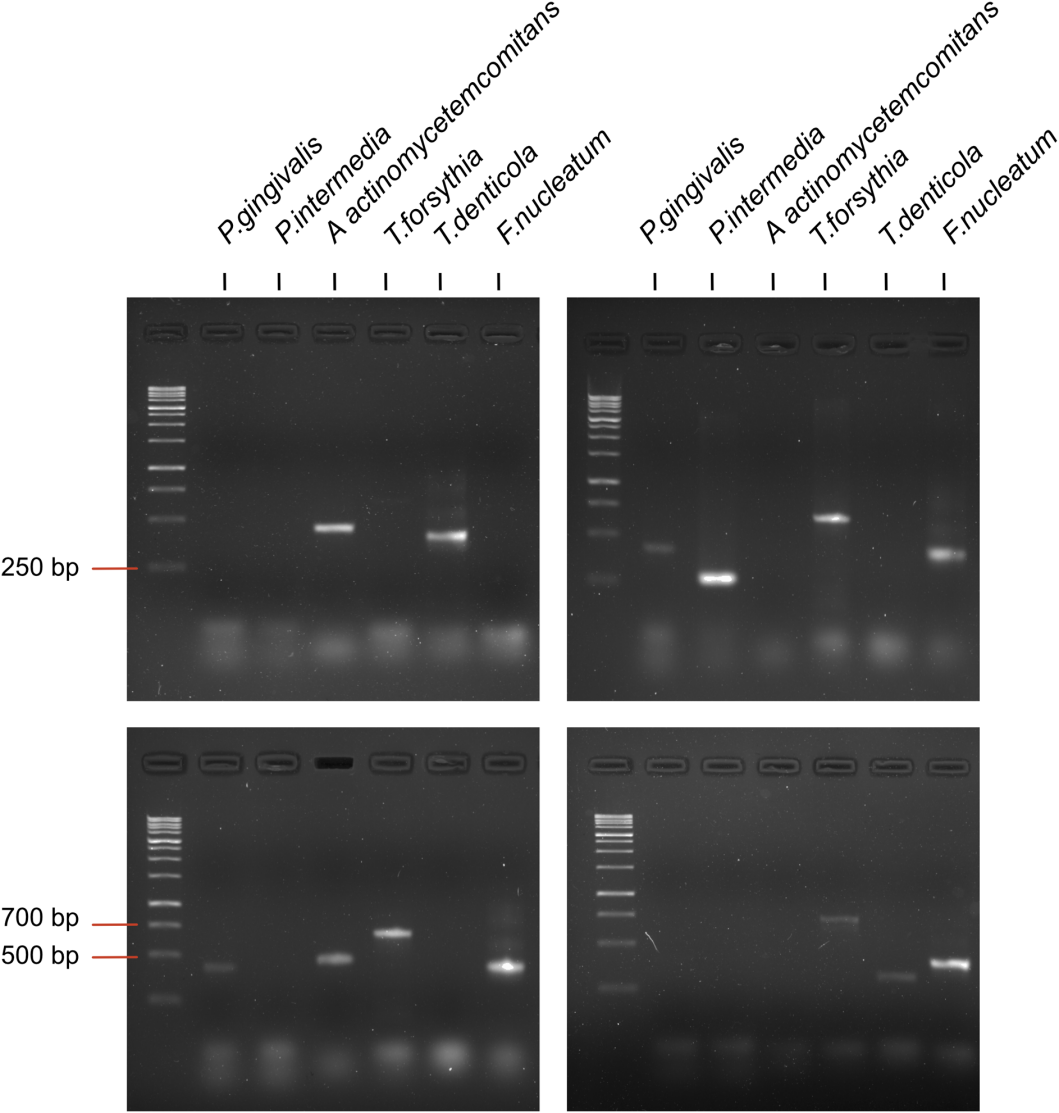
Examples of amplification of species specific amplicons of periodontal pathogenic bacteria by PCR.

For PCR, the prepared reaction mixture was used with the 5Х qPCRmix-HS (Evrogen, Russia); amplification was performed using a BioRad iQ cycler (Bio-Rad, Hercules, CA, USA). The reaction pattern was as follows: primary denaturation at 95°C for 10 min; denaturation at 95°C for 30 s; primer annealing at 60°C–68°C for 40 s; elongation at 72°C for 45 s (40 cycles). PCR products were separated by electrophoresis on 2% agarose gel (Figure 2) and analyzed with Gel Doc XP Workstation (Bio-Rad, USA). Due to the insufficient DNA concentration present in several of the analyzed samples, it was not possible to obtain PCR amplification results for all targeted bacteria (*P. gingivalis n* = 182, *P. intermedia n* = 182, *A. actinomycetemcomitans n* = 175, *T. forsythia n* = 176, *T. denticola n* = 176, *F. nucleatum n* = 176).

### 2.7 Statistics analysis

The normal distribution of the data was assessed using the Kolmogorov-Smirnov test. A chi-square test was used to calculate the frequency distributions, and a non-parametric Mann–Whitney *U*-test was used to calculate the differences in continuous variables between conception outcomes. Associations between the presence of periodontal bacteria in oral samples and the main characteristics of the participants, biochemical parameters, and calculated SCORE and FINDRISC indices were performed using Tau-b-Kendall's correlation analysis. A *p*-value of <0.05 was considered to be statistically significant. IBM SPSS Statistics v22 (IBM Corp., Armonk, NY, USA) was used for all calculations. Set analysis and Venn diagram construction were performed using the InteractiVenn web tool (40).

## 3 Results

### 3.1 Clinical characteristics of the study groups

The analysis of demographics and chronic diseases prevalence was conducted. The findings of the comparative analysis by cohort are presented in Table 2. No significant gender differences were found. Furthermore, no notable age variance was observed between the two age groups. In contrast to the young control group, the young obese group had hypertension (63.1%), dyslipidemia (38.5%), and nonalcoholic fatty liver disease (NAFLD) (35.1%) as significantly prevalent. Additionally, a modest increase in the prevalence of smoking was observed among individuals in the young obese group (35.0% vs. 27.1%). For older individuals, the differences in the prevalence of chronic diseases were considerably less pronounced. The Older Obese group included more participants with NAFLD and more smokers.

**Table 2:** The baseline characteristics of study groups (categorical parameters).

Parameter	Young individuals			Older individuals			*p* value^a^	*p* value^b^
	Young obese (*n* = 57)	Young control (*n* = 48)	*p* value	Older obese (*n* = 57)	Older control (*n* = 27)	*p* value		
Gender, *N* (%)	M—18 (31.6) F—39 (68.4)	M—13 (27.1) F—35 (72.9)	0.670	M—10 (17.5) F—47 (82.5)	M—3 (11.1) F—23 (88.9)	0.480	0.083	0.052
Age [years]	32 [28;39]	28 [26;34.7]	0.061	64 [62;67]	68 [62;74]	0.059	**0** **.** **001**	**0**.**001**
Hypertension, *N* (%)	36 (63.1)	0 (0)	**0**.**001**	52 (91.2)	22 (81.4)	0.410	**0**.**001**	**0**.**001**
Chronic heart failure, *N* (%)	1 (1.7)	0 (0)	0.112	31 (54.4)	11 (40.7)	0.950	**0**.**001**	**0**.**001**
Glucose Intolerance, *N* (%)	8 (14)	1 (2.1)	**0**.**018**	14 (24.5)	3 (11.1)	0.360	0.373	**0**.**001**
Type 2 diabetes, *N* (%)	4 (7.0)	0 (0)	0.74	13 (22.8)	4 (14.8)	0.600	**0**.**006**	**0**.**001**
Dyslipidemia, *N* (%)	22 (38.5)	0 (0%)	**0**.**001**	43 (75.4%)	15 (55.5)	0.600	**0**.**001**	**0**.**001**
NAFLD, *N* (%)	20 (35.1)	0 (0)	**0**.**001**	28 (49.2)	5 (18.5)	**0**.**011**	0.239	**0**.**001**
CAD, *N* (%)	5 (8.7)	0 (0)	**0**.**05**	19 (33.3)	8 (29.6)	0.450	**0**.**004**	**0**.**001**
Current smoking, *N* (%)	20 (35.0)	13 (27.1)	**0**.**043**	10 (17.5%)	2 (7.4%)	**0.045**	0.114	0.130

*p* values ≤0.05 are shown in bold.

^a^ Comparative analysis was conducted between the young obese and older obese groups.

^b^ Comparative analysis was conducted between the young control and older control groups.

The findings indicated a correlation between elevated blood pressure levels and the presence of obesity among both younger and older participants. The data highlighted that blood pressure values (sBP, dBP, mBP) were dependent on obesity and age. Blood pressure was higher in both obese and older participants. However, the age of the participants appeared to exert a greater influence on blood pressure than BMI. The increase in age was generally characterized by an increase in fat, including visceral fat, and a decrease in muscle mass and basal metabolic rate. The data is presented in Table 3.

**Table 3:** The baseline characteristics of study groups (continuous parameters). Data are presented as median and interquartile range.

Parameter	Young individuals			Older individuals			*p* value^a^	*p* value^b^
	Young obese (*n* = 57)	Young control (*n* = 48)	*p* value	Older obese (*n* = 57)	Older control (*n* = 27)	*p* value		
sBP, mmHg	125 [120;140]	120 [112;120]	**0** **.** **001**	140 [130;150]	130 [130;140]	**0**.**023**	**0**.**003**	**0**.**001**
dBP, mmHg	80 [80;90]	80 [70;80]	**0**.**001**	90 [80;90]	80 [80;90]	**0**.**008**	**0**.**017**	**0**.**001**
mBP, mmHg	95.0 [93.3;106.7]	93.3 [87.5;93.3]	**0**.**001**	106.7 [96.7;111.6]	96.7 [93.3;106.7]	**0**.**015**	**0**.**001**	**0**.**001**
Height, cm	170 [165;178]	174 [167;180]	0.097	163 [157;168]	162 [159;167]	0.790	**0**.**001**	**0**.**001**
Body weight, kg	102 [86.7;131.1]	65 [57.2;71.0]	**0**.**001**	100.0 [92;121.5]	70 [65;74]	**0**.**001**	0.258	0.080
BMI, kg/m^2^	35.1 [31.2;42.1]	21.2 [20.0;22.9]	**0**.**001**	37.8 [34.5;43.6]	26.2 [24.6;28.4]	**0**.**001**	0.143	0.070
Fat mass, kg	42.2 [34.3;57.8	14.9 [11.9;19.0]	**0**.**001**	47.7 [41.1;57.4]	27.0 [23.6;30.2]	**0**.**001**	0.920	**0**.**010**
Visceral adipose tissue area, cm^2^	200.0 [166.3;238.7]	65.8 [51.1;84.2]	**0**.**001**	242.2 [211.5;266.5]	141.4 [125.6;155.9]	**0**.**001**	**0**.**002**	**0**.**010**
Muscle mass, kg	32.9 [27.9;40.7]	27.0 [23.0;31.6]	**0**.**001**	28.1 [25.6;32.1]	24.6 [22.2;25.7]	**0**.**001**	0.920	**0**.**010**
Fat-to-muscle ratio	1.3 [1.0;1.6]	0.6 [0.4;0.7]	**0**.**001**	1.7 [1.4;1.9]	1.2 [1.0;1.3]	**0**.**001**	**0**.**001**	**0**.**001**
Total body water, L	45.2 [37.3;51.1]	36.6 [31.0;43.5]	**0**.**001**	37.2 [34.4;41.9]	32.8 [30.2 ± 34.5]	**0**.**001**	**0**.**001**	**0**.**005**
Basal metabolic Rate, kcal	1,629 [1,458;1,899]	1,485 [1,291;1,674]	**0**.**001**	1,464 [1,387;1,586]	1,332 [1,258;1,385]	**0**.**001**	**0**.**010**	**0**.**010**

*p* values ≤0.05 are shown in bold.

^a^ Comparative analysis was conducted between the young obese and older obese groups.

^b^ Comparative analysis was conducted between the young control and older control groups.

Furthermore, significant differences were observed in biochemical parameters between the cohorts. The young obese group exhibited elevated alanine aminotransferase (ALT) values, although these remained within the normal range. Additionally, higher uric acid levels, along with altered lipid metabolism parameters [total cholesterol (TC), triglycerides (TG), and low-density lipoproteins (LDL)], and lower high-density lipoprotein (HDL) levels were observed. Among the elderly obese participants, a similar trend was noted, with elevated uric acid levels and reduced HDL concentrations in plasma. The appropriate cardiovascular risk assessment criteria were employed for the various age groups. It was found that obesity was a significant contributor to the observed increases in both cardiovascular risk (SCORE) and diabetes risk (FINDRISC) indexes. The Older Obese group was dominated by participants at moderate and high risk on the SCORE scale, while the Older Control group was dominated by participants at moderate risk. According to the FINDRISC scale, the young obese group had a higher proportion of moderate-risk individuals, while the young control group had a higher proportion of low-risk individuals; the obese older adults had a higher proportion of high-risk participants, while the non-obese group had a higher proportion of moderate-risk participants. The data is presented in Table 4.

**Table 4:** Comparison of biochemical parameters and CVD and diabetes risk indexes between the study groups. Data are presented as median and interquartile range.

Parameter	Young individuals			Older individuals			*p* value^a^	*p* value^b^
	Young obese (*n* = 57)	Young control (*n* = 48)	*p* value	Older obese (*n* = 57)	Older control (*n* = 27)	*p* value		
AST, U/L	20.0 [17.7;30.4]	18.9 [16.5;20.8	0.100	21.5 [18.9;26.8]	26.5 [22.3;29.2]	0.100	0.312	**0** **.** **050**
ALT, U/L	22.1 [18.0;39.5]	15.0 [12.0;20.0]	**0**.**001**	19.0 [15.5;25.0]	20.0 [14.5;27.2]	0.940	**0**.**050**	0.104
Uric acid, µmol/L	353.1 [281.7;423.0]	263.8 [221.1;292.7]	**0**.**001**	376.5 [331.0;427.3]	271.9 [232.7;315.8]	**0**.**001**	0.218	0.536
Glucose, mmol/L	5.2 [4.6;5.4]	4.8 [4.6;5.0]	0.190	5.4 [4.8;6.0]	5.1 [4.8;5.5]	0.240	**0**.**009**	0.070
TC, mmol/L	5.0 [4.2;5.6]	4.5 [4.1;5.0]	**0**.**044**	5.3 [4.4;6.2]	5.7 [4.5;6.5]	0.460	0.115	**0**.**010**
TG, mmol/L	1.1 [0.8;1.5]	0.8 [0.6;1.0]	**0**.**001**	1.5 [1.1;2.1]	1.2 [1.0;1.7]	0.10	**0**.**002**	**0**.**001**
LDL cholesterol, mmol/L	3.3 [2.7;3.9]	2.6 [2.4;3.1]	**0**.**001**	3.3 [2.5;4.2]	3.4 [2.5;4.3]	0.930	0.866	**0**.**008**
HDL cholesterol, mmol/L	1.2 [0.9;1.3]	1.5 [1.2;1.7]	**0**.**001**	1.1 [1.0;1,4]	1.5 [1.3;1.7]	**0**.**001**	0.811	0.545
LDL/HDL ratio	0.4 [0.3;0.5]	0.6 [0.4;0.6]	**0**.**001**	0.4 [0.3;0.4]	0.4 [0.4;0.6]	**0**.**003**	0.782	0.056
Non-HDL mmol/L	3.8 [3.0;4.3]	3.1 [2.7;3.5]	**0,001**	4.1 [3.2;5.0]	4.1 [3.0;4.7]	0.469	0.063	**0.003**
TG/HDL ratio	1.0 [0.7;1.4]	0.6 [0.4;0.7]	**0**.**001**	1.3 [0.9;2.0]	0.9 [0.6;1.2]	**0**.**002**	**0**.**007**	**0**.**004**
TG/LDL ratio	0.4 [0.3;0.5]	0.3 [0.2;0.4]	**0**.**013**	0.5 [0.3;0.6]	0.4 [0.3;0.4]	**0**.**022**	**0**.**001**	**0**.**015**
Atherogenic coefficient	3.2 [2.6;4.1]	2.1 [1.8;2.5]	**0**.**001**	3.5 [2.7;4.6]	2.7 [2.0;3.3]	**0**.**002**	0.143	**0**.**017**
IL-1β, pg/ml	5.5 [4.5;8.5]	7.5 [4.7;10.2]	0.068	6.0 [5.0;8.0]	5.5 [5.0;7.5]	0.750	0.223	0.830
IL-6, pg/ml	1.6 [1.0;2.7]	2.0 [1.0; 3.2]	0.10	1.6 [1.0;2.0]	1.4 [1.0;2.2]	0.480	0.592	0.567
TNF-α, pg/ml	22.0 [14.0;29.0]	17.5 [12.0;27.2]	0.42	24.0 [15.5;27.5]	23.0 [13.0;25.5]	0.095	0.133	0.592
Relative CVD risk	2 [1;2]	1 [1;1]	**0**.**001**					
SCORE				4 [3;6]	3 [2;4]	**0**.**001**		
FINDRISC	11.0 [8;12.5]	3 [2;4]	**0**.**001**	16 [13;20]	11 [10;13.5]	**0**.**001**	**0**.**001**	**0**.**001**

*p* values ≤0.05 are shown in bold.

^a^ Comparative analysis was conducted between the young obese and older obese groups.

^b^ Comparative analysis was conducted between the young control and older control groups.

A number of oral health parameters were evaluated using self-reported data, including the prevalence of bruxism, the incidence of bleeding on brushing, the prevalence of dentin hypersensitivity, the use of dentures, and the frequency of dental visits. No significant differences were identified between the groups of young individuals with and without obesity. However, a trend towards a decrease in the frequency of dental visits was observed in the young obese group. No differences were identified in the group of elderly participants. Conversely, an increase in the prevalence of bleeding on brushing, use of dentures, and frequency of dental visits was observed when comparing young and elderly individuals (Table 5).

**Table 5:** Comparison of several dental self-reported parameters between the study groups.

Parameter	Young individuals			Older individuals			*p* value^a^	*p* value^b^
	Young obese (*n* = 57)	Young control (*n* = 48)	*p* value	Older obese (*n* = 57)	Older control (*n* = 27)	*p* value		
Bruxism, *N* (%)	8 (14.0)	5 (10.4)	0.710	9 (15.7)	4 (14.8)	0.760	0.960	0.940
Bleeding on brushing, *N* (%)	18 (31.5)	13 (27.1)	0.580	31 (54.3)	12 (44.4)	0.560	**0** **.** **030**	0.070
Dentin hypersensitivity *N* (%)	18 (31.5)	18 (37.5)	0,540	20 (35.1)	9 (33.3)	0.920	0.750	0.790
Use of dentures *N* (%)	4 (7.1)	3 (6.2)	0,320	31 (54.3)	18 (66.7)	0.720	**0**.**010**	**0**.**010**
Dental visits, *N* (%)								
Every 6 month	10 (17.5)	11 (22.9)	0.080	3 (5.3)	3 (11.1)	0.240	**0**.**030**	**0**.**020**
Every 1 year	14 (24.6)	19 (39.6)		11 (19.3)	11 (40.7)			
Every 2–3 years	25 (43.9)	13 (27.1)		21 (36.8)	6 (22.2)			
Every 3 + years	8 (14.0)	5 (10.4)		22 (38.5)	7 (26.0)			

*p* values ≤0.05 are shown in bold.

^a^ Comparative analysis was conducted between the young obese and older obese groups.

^b^ Comparative analysis was conducted between the young control and older control groups.

### 3.2 The prevalence of the periodontal pathogens in the study groups

**Figure 3:**
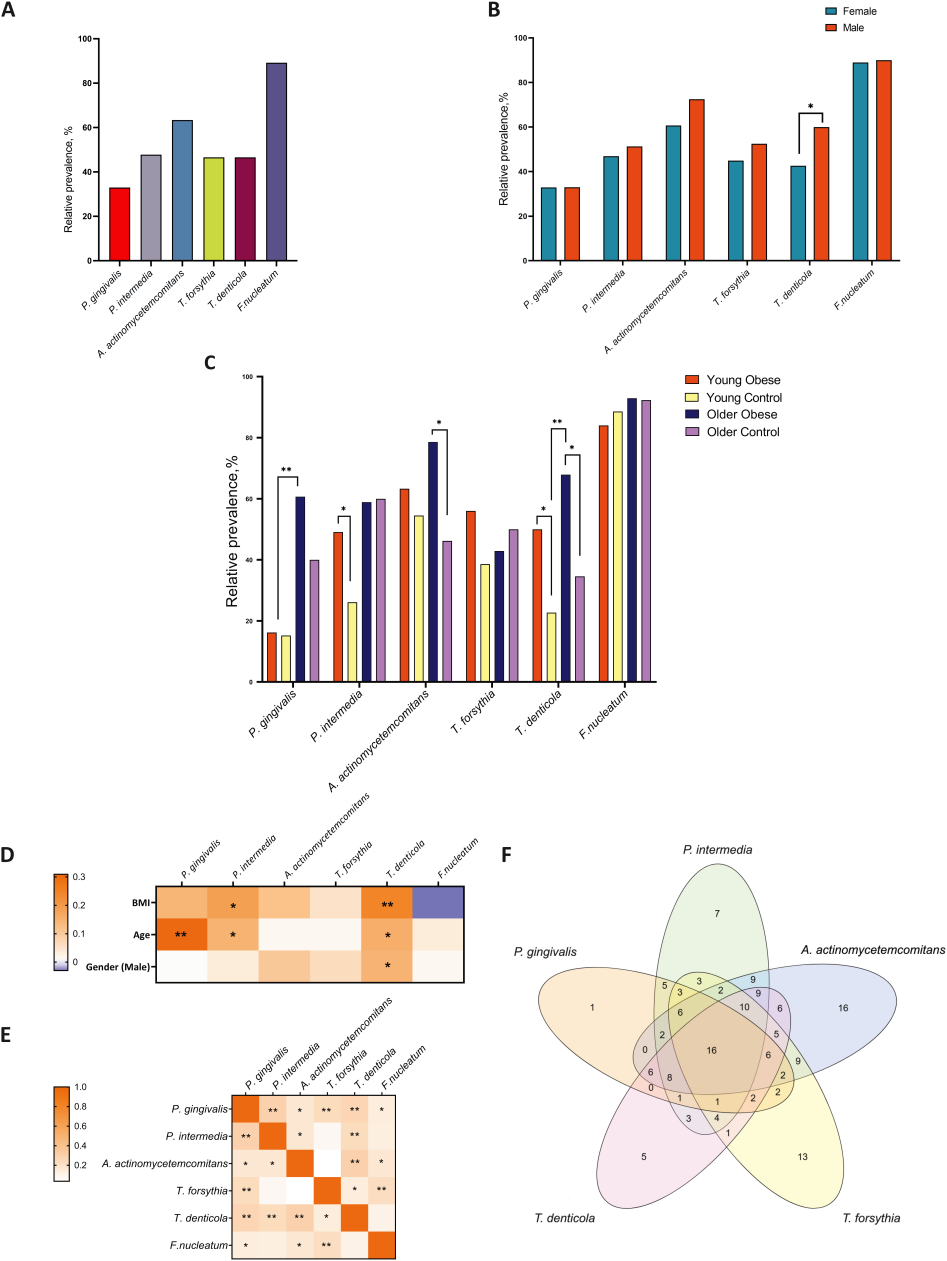
The relative prevalence of major periodontal pathogens among all participants **(A)** compared by gender **(B)** and in the study groups **(C)** correlations between periodontal pathogens and age, gender, and BMI **(D)** correlation analysis for interbacterial associations **(E)** the Venn diagram is based on the analysis of the concordance of the prevalence of five microorganisms in the overall population **(F)** * *p* ≤ 0.05; ** *p* ≤ 0.01.

The prevalence of periodontal pathogens among participants was investigated using polymerase chain reaction (PCR) analysis. The overall prevalence of periodontal pathogens in the study population was 33.0% for *P. gingivalis*, 47.8% for *P. intermedia*, 63.4% for *A. actinomycetemcomitans*, 46.6% for *T. forsythia*, 46.6% for *T. denticola*, and 89.2% for *F. nucleatum* (Figure 3A). The gender differences were only confirmed for T. denticola (Figure 3B). This species was more prevalent in males (60.0% vs. 42.3%, *p* = 0.047). The prevalence of *P. gingivalis* did not differ significantly between the young obese and young control groups (16.4% vs. 15.2% *p* = 0.635). However, in older adults with obesity, the prevalence tended to be higher compared to non-obese individuals (60.0% vs. 40.0%, *p* = 0.084). The data revealed a significant effect of age on the prevalence of this species. Significant differences were observed in the prevalence of *P. intermedia* among younger participants, with a greater proportion in obese individuals (49.1% vs. 26.1%, *p* = 0.019). However, no differences were observed between the older age groups (58.9% vs. 60.0%, *p* = 0.928). *A. actinomycetemcomitans* was detected to be more prevalent among the older cohort of obese participants than in non-obese participants (78.6% vs. 46.2%. *p* = 0.004). *T. forsythia* was found to tend to be more common in young obese subjects, while no significant difference was identified. The occurrence of *T. denticola* exhibited a stronger correlation with age than BMI. The prevalence was 50.0% and 22.7% (*p* = 0.007) for the Young Obese and Young Control groups and 67.9% and 34.6% (*p* = 0.005) for the Older Obese and Older control groups, respectively. *F. nucleatum* was identified in nearly all the samples and was found to be independent of weight or age (Figure 3C).

Furthermore, a set analysis was conducted to evaluate the prevalence of five specific microorganisms (*P. gingivalis*, *P. intermedia*, *A. actinomycetemcomitans*, *T. forsythia*, and *T. denticola*) within the entire population. The presence of five bacteria was identified in 13 (6.8%) participants. At the same time, a single bacterium was identified in the samples of 1, 8, 16, 12, and 5 participants, respectively (Figure 3F). All 6 bacteria were found in 12 (6.3%) participants, while none of the bacteria were identified in 16 (8.5%). The results of the correlation analysis indicated the presence of notable interactions between the species *P. gingivalis* and *P. intermedia*, as well as between *T. forsythia* and *T. denticola*. *P. intermedia* is associated with *T. denticola*, and *A. actinomycetemcomitans* is associated with *T. denticola* as well. Additionally, there is a notable correlation between *T. forsythia* and *F. nucleatum* (Figure 3E).

### 3.3 The relationship between periodontal pathogens and CVD risk factors

**Figure 4:**
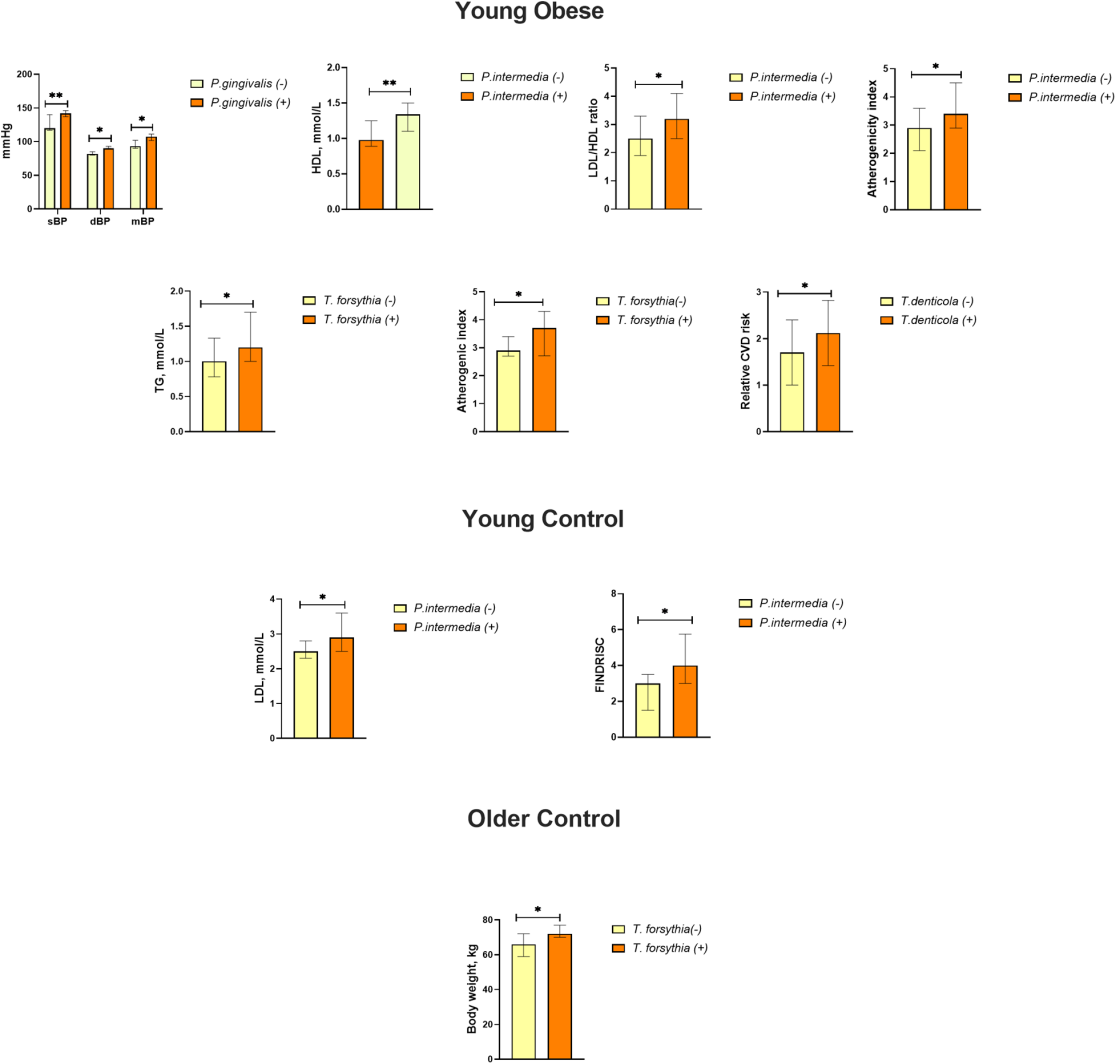
Effects of the presence of periodontal bacteria on blood pressure, lipid metabolism, body weight, and CVD risk (relative CVD risk, SCORE) and FINDRISC scores in the study groups. Data are presented as median and interquartile range. * *p* ≤ 0.05; ** *p* ≤ 0.01.

This study examined the link between periodontal pathogens and major risk factors for cardiovascular disease. A comparison of participants with and without periodontal pathogens revealed significant differences (Figure 4). The group of young obese individuals exhibited the highest number of parameters indicative of exposure to bacteria. Specifically, individuals with detected *P. gingivalis* demonstrated higher sBP (140 [137;146] mmHg vs. 120 [117;140], *p* = 0.001 mmHg, dBP 90 [85;93] mmHg vs. 82 [80;85] mmHg, *p* = 0.005 and MBP 107 [101;111] mmHg vs. 93 [90;102] mmHg, *p* = 0.002). The results demonstrated that *P. intermedia* was associated with lower HDL levels [0.98 [0.89;1.25] mmol/L vs. 1.34 [1.1;1.5] mmol/L, *p* = 0.001] and higher LDL/HDL ratios [3.2 [2.5;4.1] vs. 2.5 [1.9;3.3], *p* = 0.029] and atherogenic index [3.4 [2.9;4.5] vs. 2.9 [2.1;3.6], *p* = 0.047]. The presence of *T. forsythia* was associated with elevated triglyceride levels [1.2 [1.0;1.7] mmol/L vs. 1.0 [0.8;1.3] mmol/L, *p* = 0.028] and an increased atherogenic index [3.7 [2.7;4.3] vs. 2.9 [3.4;2.7], *p* = 0.036]. A positive link was observed between the presence of *T. denticola* and the Relative CVD risk (2.1 ± 0.7 vs. 1.7 ± 0.7, *p* = 0.031). Furthermore, in the young control group, *P. intermedia* was associated with higher LDL [2.9 [2.5;3.6] mmol/L vs. 2.5 [2.3;2.8] mmol/L, *p* = 0.039] and FINDRISC score [4 [3;6] vs. 3 [2;4], *p* = 0.043], although overall these values remained within the normal range. The prevalence of *T. forsythia* among older control participants was associated with higher body weight [72 [70;77] kg vs. 66 [59;72] kg, *p* = 0.048], but not with BMI (Figure 4).

**Figure 5:**
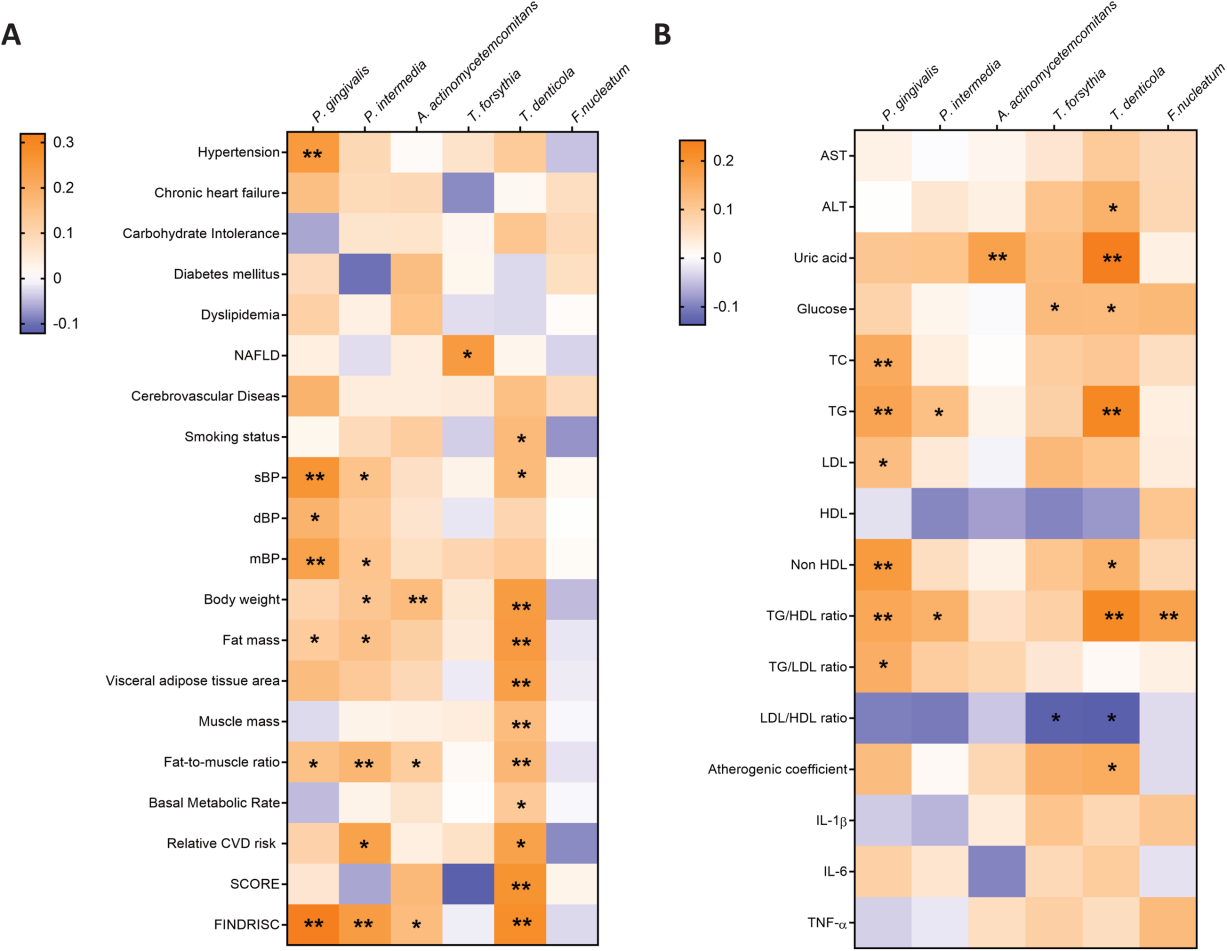
The Tay-b-kendall's correlation analysis was conducted to investigate the associations between periodontal pathogens and baseline participant characteristics **(A)** and biochemical parameters **(B)** among all study participants. * *p* ≤ 0.05; ** *p* ≤ 0.01.

The Tau-b-Kendall's correlation analysis was also conducted in order to identify some potential associations between periodontal pathogens and risk factors among all study participants (Figures 5A,B). Among all established diagnoses, only *P. gingivalis* was found to be significantly associated with hypertension and *T. forsythia* with NAFLD. A positive correlation was observed between *T. denticola* and smoking status. As observed in the young obese cohort, *P. gingivalis* was found to be correlated with all blood pressure parameters. Additionally, *P. intermedia* was associated with elevated sBP and mBP among all participants. The presence of *P. intermedia* and *T. denticola* was connected with higher SCORE in the younger cohort, whereas only *T. denticola* was linked to raised SCORE in the older group. Furthermore, a positive correlation was also indicated between FINDRISC and the presence of *P. gingivalis, P. intermedia, A. actinomycetemcomitans* and *T. denticola*. A positive correlation between periodontal bacteria and body composition parameters, particularly body weight, fat mass, and visceral fat area was also revealed. The strongest correlation with these parameters was observed for *T. denticola.* However, it is noteworthy that muscle mass and basal metabolic rate were also high. The presence of periodontal pathogens was also related to higher serum levels of TC, LDL, TG, glucose and lower levels of HDL and the LDL/HDL ratio in general. *P. gingivalis* was positively associated with TC, TG, and LDL. *T. forsythia* was associated only with LDL, while *T. denticola* was linked to TG. At the same time, *T. forsythia* and *T. denticola* were linked to elevated glucose levels. Additionally, higher uric acid levels were correlated with the presence of *A. actinomycetemcomitans* and *T. denticola*. Proinflammatory cytokine levels were not significantly associated with any periodontal pathogen in our study.

## 4 Discussion

The oral microbiome is the second largest in terms of species and total number of microorganisms after the intestinal microbiome (41). A number of opportunistic bacteria are responsible for the development of such widespread diseases as periodontitis and caries, and can have a systemic effect on the body (21). The objective of this study was to assess the prevalence of six major periodontal pathogens according to age (young/old), gender, and obesity. In addition to the impact of microorganisms on CVD risk factors, including smoking, obesity, lipid and carbohydrate metabolism indicators, and SCORE and FINDRISC scales.

Overall, our findings indicate that both age and obesity are associated with a higher prevalence of periodontal pathogens. Specifically, *P. gingivalis* was more prevalent in obese and elderly individuals, while no significant difference was observed among young individuals. The prevalence of *P. intermedia* was higher in the young obese subjects compared to the control young group, while *A. actinomycetemcomitans* was more prevalent in the elderly obese subjects compared to the elderly non-obese. The prevalence of *T. denticola* was dependent on the BMI, as observed in both young and old individuals. A gender difference was found only for *T. denticola*, which was more frequent in males. The study demonstrated a comparable prevalence of *P. gingivalis* and *P. intermedia*.

 According to the literature, the incidence of *A. actinomycetemcomitans*, *F. nucleatum*, and *T. forsythia* was markedly diminished in patients without type 2 diabetes. Furthermore, *P. gingivalis* was identified with greater frequency in overweight patients with type 2 diabetes mellitus (42). Another study revealed that adults over the age of 35 exhibited a higher prevalence of *A. actinomycetemcomitans*, whereas *T. forsythia* was more prevalent in younger adults. In addition, the prevalence of *T. denticola* differed by gender among the various bacterial species, with a higher prevalence observed in men (43). A study conducted in the Middle East and North African population without advanced periodontitis revealed a higher prevalence of *P. gingivalis* and *P. intermedia*, and a lower prevalence of *A. actinomycetemcomitans*. There was a statistically significant association between *P. gingivalis* and *A. actinomycetemcomitans*. There was no reliable correlation between *P. intermedia* and *A. actinomycetemcomitans* (44)*.* In individuals from the Slovak population with periodontitis, a higher prevalence of *T. denticola*, *P. gingivalis*, *T. forsythia*, and *A. actinomycetemcomitans* was observed, with *P. gingivalis* being present in 100% of cases (45). It is noteworthy that another study identified a marginally lower overall prevalence of *P. gingivalis* and an inverse correlation with age, in addition to demonstrating the influence of ethnicity (46). The principal mechanism that may be accountable for the observed increase in the prevalence of oral diseases and the invasion of pathogenic microorganisms with age may be a reduction in the activity of innate immunity (47). Gender differences in the prevalence of oral microorganisms may be attributed to a number of factors, including a tendency for men to exhibit poorer oral hygiene and less frequent dental visits, as well as the potential influence of hormonal levels (48, 49). Furthermore, a notable correlation was identified between smoking status and the presence of *T. denticola* in the general population. The extant literature is inconclusive, with one study of 60 individuals indicating that smoking was associated with a higher prevalence of *T. denticola* and also with a suppressed inflammatory response (50). The findings of another study examining the effects of electronic cigarettes did not indicate a similar correlation (51).

The interaction of microorganisms with each other in the composition of coaggregates or biofilms plays a crucial role in providing their pathogenic effect (52). Moreover, opportunistic pathogens may be present in healthy individuals with intact periodontium (53). This study showed a correlation between the presence of periodontal pathogens. *P. gingivalis* was more frequently observed in the presence of *P. intermedia* and *T. denticola*. Similarly, *P. intermedia* and *A. actinomycetemcomitans* were more often detected in the oral cavity alongside *T. denticola*, while *T. forsythia* and *F. nucleatum* were commonly found together. A recent study showed that co-occurrence patterns may vary depending on the presence and severity of oral disease. For example, in individuals with healthy periodontium, *P. gingivalis* was more likely to co-occur with *P. intermedia*, whereas in periodontitis, *P. gingivalis* was associated with *T. denticola* and *T. forsythia*. *T. forsythia* was also found together with *F. nucleatum* (54). Furthermore, *Fusobacteria*, including *F. nucleatum*, are thought to bind early and late colonizers in dental plaque. The expression of galactose-specific lectin allows it to bind to *P. gingivalis* (55). Other studies have found that *P. gingivalis* stimulates the growth of *T. denticola* through the production of isobutyric acid, folate, and glycine. In turn, *T. denticola* produces succinic acid, which serves to enhance the growth of *P. gingivalis* (56, 57).

This study examined the relationship between the presence of periodontal pathogens and hypertension. The most significant association was found between hypertension and *P. gingivalis*, both at the level of diagnosis and at the level of sBP, dBP and mBP. Notably, when the study groups were considered, the difference was significant only in young adults with obesity. A positive correlation between high blood pressure and the presence of *P. intermedia* and *T. denticola* also found in the general population. It is established that the oral microbiome exerts an influence on blood pressure via its capacity to serve as an autonomous source of nitric oxide (NO), operating independently of the nitric oxide synthase (NOS) pathway (58). A variety of bacterial species are capable of producing nitric oxide in the oral cavity (59). The most extensively researched factor contributing to the maintenance of normal blood pressure is a relatively high level of *Neisseria subflava* and *Corynebacterium durum* in saliva. A notable decline in concentration was observed in individuals with hypertension (60, 61). A study of 653 participants demonstrated an association between elevated levels of the periodontal pathogens *P. gingivalis*, *T. forsythia*, *A. actinomycetemcomitans*, *T. denticola* and hypertension (62). Nevertheless, the precise mechanisms by which these bacteria may affect vascular tone remain unclear. A study conducted on C57BL/6J mice demonstrated that *P. gingivalis* may facilitate a reduction in angiotensin II levels (63).

The study did not reveal any notable correlation between the prevalence of periodontal pathogens and the incidence of obesity in the examined groups. Nevertheless, in the overall population, the most notable discrepancy was observed with the presence of *T. denticola*. Individuals who had this species exhibited differences in greater body weight, BMI, visceral fat area and a higher fat/muscle ratio. Furthermore, the findings indicated a correlation between *P. intermedia* and elevated BMI and fat mass across the entire study population. Data on the relationship between periodontal bacterial overgrowth and obesity are controversial. A recent study demonstrated a correlation between the presence of *A. actinomycetemcomitans* and obesity, with *T. forsythia* and *T. denticola* also identified in overweight individuals. In contrast, *P. gingivalis* and *F. nucleatum* were observed exclusively in those with a normal weight (64). Moreover, evidence suggests an association between an increased prevalence of *F. nucleatum* and *P. intermedia* in obese patients with periodontitis compared to those with a healthy metabolic profile (65). A study of 695 subjects demonstrated a correlation between the overgrowth of *T. forsythia* and the prevalence of overweight and obesity in individuals with a healthy periodontium (66). In another study, *T. forsythia* was demonstrated to be a contributing factor in the formation of a yellow coating on the tongue and to enhance the perception of taste for fatty foods (67).

**Figure 6:**
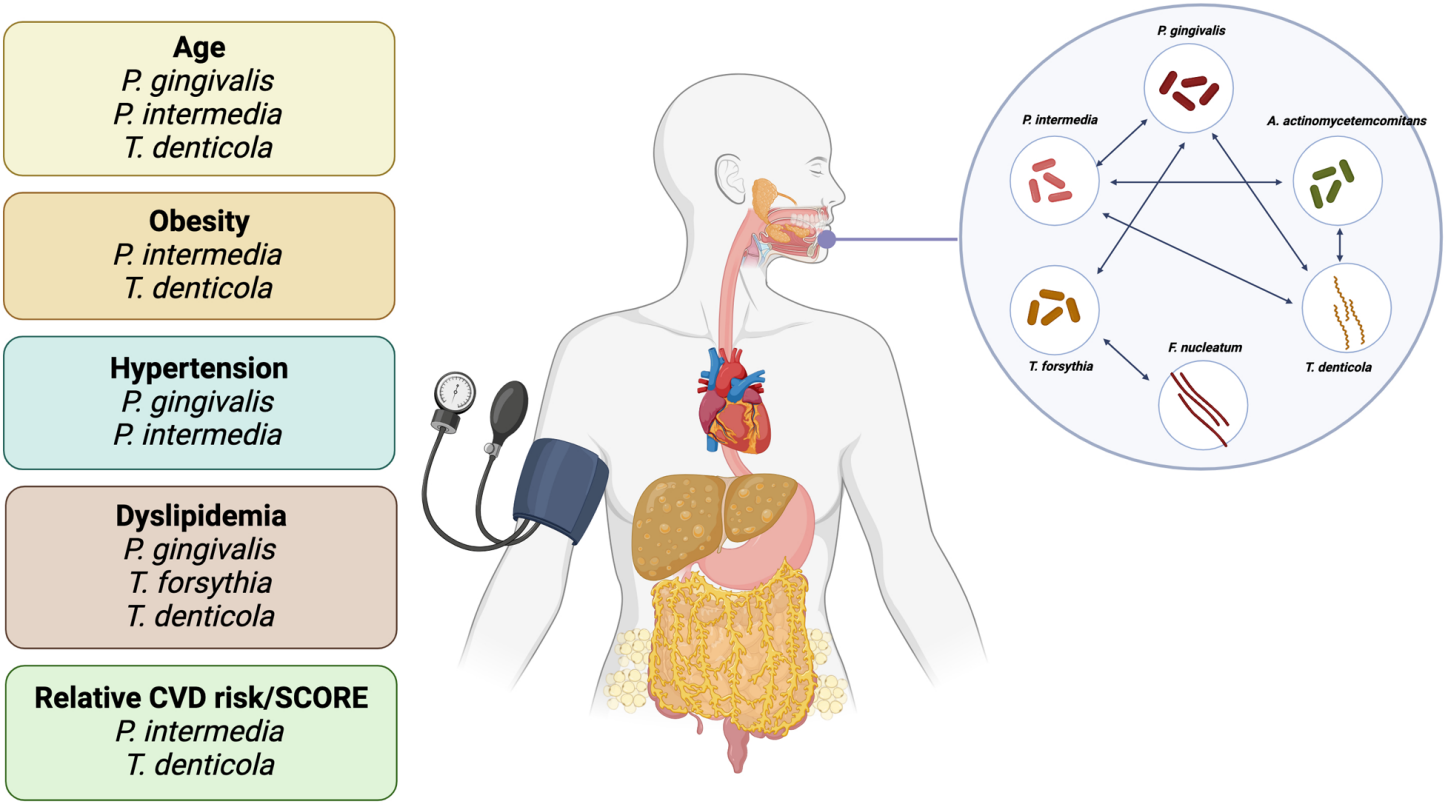
The relationships between periodontal pathogenic bacteria and factors such as age, obesity, hypertension, dyslipidemia and CVD risk are examined. Furthermore, the primary correlations between bacteria are presented.

A correlation was identified between lipid metabolism parameters and the presence of specific oral microorganisms, including *P. gingivalis*, *P. intermedia*, *T. forsythia* and *T. denticola*. It is noteworthy that the most significant differences were observed among the young obese group. Thus, HDL levels were found to be lower in individuals positive for *P. intermedia*, and the presence of *T. forsythia* was associated with higher LDL levels. In the overall population, serum concentrations of TC, LDL, and TG were found to positively correlate with the presence of *P. gingivalis*. Furthermore, a significant positive association was identified between *T. denticola* and TG levels (Figure 6). A meta-analysis comprising 29 studies demonstrated a connection between periodontitis and dyslipidemia. In particular, TC, LDL, and TG levels were significantly elevated in individuals with periodontitis, while HDL levels were reduced (68). Simultaneously, it was demonstrated that patients affected with periodontitis and dyslipidemia exhibited elevated incidences of bleeding on probing (BOP) and clinical attachment loss (CAL) (69). In an *in vivo* model of periodontitis induced by *A. actinomycetemcomitans* and *P. gingivalis*, it was demonstrated that a high-fat diet-induced dyslipidemia was associated with a notable elevation in systemic inflammation and bone loss (70). Another study in apolipoprotein E-deficient (ApoE^−/−^) mice showed that dyslipidemia impairs the innate immune response to *P. gingivalis* challenge, which may contribute to the increased activity of this species (71). Moreover, the combination of hyperlipidemia and periodontitis, but not only periodontitis, can lead to the development of atherosclerosis (72). A recent study has shown that periodontal metabolic parameters can serve as biomarkers for lipid and carbohydrate metabolism disorders in overweight and obese individuals (73). Furthermore, evidence indicates that *P. gingivalis* is associated with increased oxidative stress and lipid peroxidation, particularly in LDL (74). It is noteworthy that the administration of statins and fibrates for the treatment of dyslipidemia has been observed to diminish the likelihood of developing chronic periodontitis (75, 76). In a separate study, treatment with atorvastatin or simvastatin was observed to result in a reduction in the concentration of proinflammatory markers in the blood (IL-6, CRP, TNF-α), as well as a decrease in periodontal indices (77, 78). A recent study demonstrated that enhanced oral hygiene and concomitant reductions in the levels of *P. gingivalis*, *T. denticola*, and *T. forsythia* led to improvements in the hyperglycemic status of patients with T2DM, especially younger patients (79). Observed data are not able to clarify the cause-effect connections: either the dislipidemia causes the oral biota changes or periodontitis leads to dyslipidemia or even both blood lipids changes and oral pathogens growth are co-founders and caused by poor diet. Nevertheless, the link between them is well established. Furthermore, a potential covariation exists between the prevalence of periodontal pathogens and obesity, given that obesity is a well-established risk factor for CVDs (80, 81). Further research in this field may encompass additional investigations into the prevalence of periodontal pathogens across diverse age groups and ethnicities, as well as larger-scale studies. It is also crucial to examine interspecies bacterial interactions within the oral cavity, employing both relative and absolute quantification techniques. Furthermore, the potential mechanisms by which pathogenic microorganisms may exert adverse effects on overall health and well-being warrant investigation, including the utilization of biomarkers such as lipopolysaccharides (LPS), antibodies to periodontal pathogens, and an expanded panel of cytokines and adipokines. Further investigation is necessary to determine the causal relationships between oral microbiome dysbiosis and systemic diseases.

## 5 Study limitations

This study did not examine in detail the presence of oral diseases such as caries or periodontitis, nor did it take into consideration of common periodontal indices such as periodontal pocket depth (PPD) and bleeding on probing (BOP), etc. This limitation is due to the therapeutic profile of the Nutrition Clinic of the Federal Research Center for Nutrition, Biotechnology and Food Safety. The objective of this study was to examine the presence of selected periodontal pathogens. However, the aim was not to quantify them. In addition, the insufficient number of men did not allow for gender-adjusted intergroup analysis.

## 6 Conclusion

The findings of this study underscore the significance of investigating the oral microbiome in the context of both oral health and systemic diseases, particularly concerning the correlation between periodontal pathogens and disorders such as obesity, dyslipidaemia, and hypertension. Another point of further research is the potential exists for obesity to serve as a connecting factor between oral dysbiosis and risk factors for CVDs. The high prevalence of these pathogens, including in young adults, underscores the potential benefits of preventive measures and early intervention. The plethora of studies revealing the systemic impact of periodontal disease highlights the necessity for prevention strategies to prioritise young adults, who are at a pivotal stage in establishing lifelong oral health habits. The collaboration between physicians and dentists is crucial in addressing these interconnected health issues. Medical professionals need to work together to ensure comprehensive care, recognizing that oral health is intrinsically linked to overall health. By integrating dental evaluations into routine medical check-ups, particularly for at-risk populations such as those with obesity or cardiovascular risk factors, healthcare providers can better manage and prevent the systemic effects of periodontal pathogens. This interdisciplinary approach is essential for mitigating the broader health implications of oral diseases and improving patient outcomes across the lifespan.

## Data Availability

The original contributions presented in the study are included in the article/Supplementary Material, further inquiries can be directed to the corresponding author.

## References

[1] Fan J Li X Yu X Liu Z Jiang Y Fang Y Global burden, risk factor analysis, and prediction study of ischemic stroke, 1990–2030. Neurology. (2023) 101:137–50. 10.1212/WNL.0000000000207336 37197995 PMC10351546

[2] Leopold JA Antman EM. Ideal cardiovascular health in young adults with established cardiovascular diseases. Front Cardiovasc Med. (2022) 9:814610. 10.3389/fcvm.2022.814610 35252395 PMC8893279

[3] Navar AM Fine LJ Ambrosius WT Brown A Douglas PS Johnson K Earlier treatment in adults with high lifetime risk of cardiovascular diseases: what prevention trials are feasible and could change clinical practice? Report of a national heart, lung, and blood institute (NHLBI) workshop. Am J Prev Cardiol. (2022) 12:100430. 10.1016/j.ajpc.2022.100430 36439649 PMC9691440

[4] Jin X Qiu T Li L Yu R Chen X Li C Pathophysiology of obesity and its associated diseases. Acta Pharm Sin B. (2023) 13:2403–24. 10.1016/j.apsb.2023.01.012 37425065 PMC10326265

[5] Leonov G Salikhova D Starodubova A Vasilyev A Makhnach O Fatkhudinov T Oral microbiome dysbiosis as a risk factor for stroke: a comprehensive review. Microorganisms. (2024) 12:1732. 10.3390/microorganisms12081732 39203574 PMC11357103

[6] Chait A den Hartigh LJ. Adipose tissue distribution, inflammation and its metabolic consequences, including diabetes and cardiovascular disease. Front Cardiovasc Med. (2020) 7:522637. 10.3389/fcvm.2020.00022 32158768 PMC7052117

[7] Poirier P Giles TD Bray GA Hong Y Stern JS Pi-Sunyer FX American Heart Association, obesity committee of the council on nutrition, physical activity, and metabolism. Obesity and cardiovascular disease: pathophysiology, evaluation, and effect of weight loss: an update of the 1997 American Heart Association scientific statement on obesity and heart disease from the obesity committee of the council on nutrition, physical activity, and metabolism. Circulation. (2006) 113:898–918. 10.1161/CIRCULATIONAHA.106.171016 16380542

[8] Wu Y Chi X Zhang Q Chen F Deng X. Characterization of the salivary microbiome in people with obesity. PeerJ. (2018) 6:e4458. 10.7717/peerj.4458 29576948 PMC5858547

[9] Schamarek I Anders L Chakaroun RM Kovacs P Rohde-Zimmermann K. The role of the oral microbiome in obesity and metabolic disease: potential systemic implications and effects on taste perception. Nutr J. (2023) 22:28. 10.1186/s12937-023-00856-7 37237407 PMC10223863

[10] Deo PN Deshmukh R. Oral microbiome: unveiling the fundamentals. J Oral Maxillofac Pathol JOMFP. (2019) 23:122–8. 10.4103/jomfp.JOMFP_304_18 31110428 PMC6503789

[11] Leonov GE Varaeva YR Livantsova EN Starodubova AV. The complicated relationship of short-chain fatty acids and oral microbiome: a narrative review. Biomedicines. (2023) 11:2749. 10.3390/biomedicines11102749 37893122 PMC10604844

[12] Radaic A Kapila YL. The oralome and its dysbiosis: new insights into oral microbiome-host interactions. Comput Struct Biotechnol J. (2021) 19:1335–60. 10.1016/j.csbj.2021.02.010 33777334 PMC7960681

[13] Min Z Yang L Hu Y Huang R. Oral microbiota dysbiosis accelerates the development and onset of mucositis and oral ulcers. Front Microbiol. (2023) 14:1061032. 10.3389/fmicb.2023.1061032 36846768 PMC9948764

[14] Santacroce L Passarelli PC Azzolino D Bottalico L Charitos IA Cazzolla AP Oral microbiota in human health and disease: a perspective. Exp Biol Med. (2023) 248:1288–301. 10.1177/15353702231187645 PMC10625343 37688509

[15] Ma D Chen B Li Y Pang X Fu Q Xiao Z Au@ag nanorods-PDMS wearable mouthguard as a visualized detection platform for screening dental caries and periodontal diseases. Adv Healthc Mater. (2022) 11:e2102682. 10.1002/adhm.202102682 34957703

[16] Bourgeois D Inquimbert C Ottolenghi L Carrouel F. Periodontal pathogens as risk factors of cardiovascular diseases, diabetes, rheumatoid arthritis, cancer, and chronic obstructive pulmonary disease—is there cause for consideration? Microorganisms. (2019) 7:424. 10.3390/microorganisms7100424 31600905 PMC6843669

[17] Carrouel F Viennot S Santamaria J Veber P Bourgeois D. Quantitative molecular detection of 19 Major pathogens in the interdental biofilm of periodontally healthy young adults. Front Microbiol. (2016) 7:840. 10.3389/fmicb.2016.00840 27313576 PMC4889612

[18] Veras EL Castro dos Santos N Souza JGS Figueiredo LC Retamal-Valdes B Barão VAR Newly identified pathogens in periodontitis: evidence from an association and an elimination study. J Oral Microbiol. (2023) 15:2213111. 10.1080/20002297.2023.2213111 37261036 PMC10228317

[19] Abdulkareem AA Al-Taweel FB Al-Sharqi AJB Gul SS Sha A Chapple ILC. Current concepts in the pathogenesis of periodontitis: from symbiosis to dysbiosis. J Oral Microbiol. (2023) 15:2197779. 10.1080/20002297.2023.2197779 37025387 PMC10071981

[20] Håheim L Lise A. Oral anaerobe bacteria—a common risk for cardiovascular disease and mortality and some forms of cancer? Front Oral Health. (2024) 55:1348946. 10.3389/froh.2024.1348946 38774039 PMC11107091

[21] Isola G Santonocito S Lupi SM Polizzi A Sclafani R Patini R Periodontal health and disease in the context of systemic diseases. Mediators Inflamm. (2023) 2023:9720947. 10.1155/2023/9720947 37214190 PMC10199803

[22] Peng X Cheng L You Y Tang C Ren B Li Y Oral microbiota in human systematic diseases. Int J Oral Sci. (2022) 14:14. 10.1038/s41368-022-00163-7 35236828 PMC8891310

[23] Celik D Kantarci A. Vascular changes and hypoxia in periodontal disease as a link to systemic complications. Pathogens. (2021) 10:1280. 10.3390/pathogens10101280 34684229 PMC8541389

[24] Leng Y Hu Q Ling Q Yao X Liu M Chen J Periodontal disease is associated with the risk of cardiovascular disease independent of sex: a meta-analysis. Front Cardiovasc Med. (2023) 10:1114927. 10.3389/fcvm.2023.1114927 36923959 PMC10010192

[25] Mozos I Malainer C Horbańczuk J Gug C Stoian D Luca CT Inflammatory markers for arterial stiffness in cardiovascular diseases. Front Immunol. (2017) 8:1058. 10.3389/fimmu.2017.01058 28912780 PMC5583158

[26] Rahimi A Afshari Z. Periodontitis and cardiovascular disease: a literature review. ARYA Atheroscler. (2021) 17:1–8. 10.22122/arya.v17i0.2362 PMC9137219 35686242

[27] Kitamoto S Kamada N. Periodontal connection with intestinal inflammation: microbiological and immunological mechanisms. Periodontol 2000. (2022) 89:142–53. 10.1111/prd.12424 35244953 PMC9018512

[28] Kannosh I Staletovic D Toljic B Radunovic M Pucar A Matic Petrovic S The presence of periopathogenic bacteria in subgingival and atherosclerotic plaques - an age related comparative analysis. J Infect Dev Ctries. (2018) 12:1088–95. 10.3855/jidc.10980 32027610

[29] Haynes WG Stanford C. Periodontal disease and atherosclerosis. Arterioscler Thromb Vasc Biol. (2003) 23:1309–11. 10.1161/01.ATV.0000087144.24654.71 12909566

[30] Kim J Kim HJ Jeon J Song T-J. Association between oral health and cardiovascular outcomes in patients with hypertension: a nationwide cohort study. J Hypertens. (2022) 40:374–81. 10.1097/HJH.0000000000003022 34670996

[31] Liu Y Li L Miao G Yang X Wu Y Xu Y Relationship between children’s intergenerational emotional support and subjective well-being among middle-aged and elderly people in China: the mediation role of the sense of social fairness. Int J Environ Res Public Health. (2022) 19:389. 10.3390/ijerph19010389 PMC8751110 35010647

[32] Flegal KM Kit BK Graubard BI. Body mass index categories in observational studies of weight and risk of death. Am J Epidemiol. (2014) 180:288–96. 10.1093/aje/kwu111 24893710 PMC4732880

[33] Schwarz PEH Li J Reimann M Schutte AE Bergmann A Hanefeld M The Finnish diabetes risk score is associated with insulin resistance and progression towards type 2 diabetes. J Clin Endocrinol Metab. (2009) 94:920–6. 10.1210/jc.2007-2427 19106274

[34] Ramírez-Vélez R Carrillo HA Correa-Bautista JE Schmidt-RioValle J González-Jiménez E Correa-Rodríguez M Fat-to-muscle ratio: a new anthropometric indicator as a screening tool for metabolic syndrome in young Colombian people. Nutrients. (2018) 10:1027. 10.3390/nu10081027 30087234 PMC6115891

[35] Kosmas CE Rodriguez Polanco S Bousvarou MD Papakonstantinou EJ Peña Genao E Guzman E The triglyceride/high-density lipoprotein cholesterol (TG/HDL-C) ratio as a risk marker for metabolic syndrome and cardiovascular disease. Diagnostics. (2023) 13:929. 10.3390/diagnostics13050929 36900073 PMC10001260

[36] Cheng W Wang L Chen S. Differences in lipid profiles and atherogenic indices between hypertensive and normotensive populations: a cross-sectional study of 11 Chinese cities. Front Cardiovasc Med. (2022) 9:887067. 10.3389/fcvm.2022.887067 35656401 PMC9152277

[37] Ouchi G Komiya I Taira S Wakugami T Ohya Y. Triglyceride/low-density-lipoprotein cholesterol ratio is the most valuable predictor for increased small, dense LDL in type 2 diabetes patients. Lipids Health Dis. (2022) 21:4. 10.1186/s12944-021-01612-8 34996463 PMC8742340

[38] Omori M Kato-Kogoe N Sakaguchi S Fukui N Yamamoto K Nakajima Y Comparative evaluation of microbial profiles of oral samples obtained at different collection time points and using different methods. Clin Oral Investig. (2021) 25:2779–89. 10.1007/s00784-020-03592-y 32975702

[39] Eberhard J Grote K Luchtefeld M Heuer W Schuett H Divchev D Experimental gingivitis induces systemic inflammatory markers in young healthy individuals: a single-subject interventional study. PLoS One. (2013) 8:e55265. 10.1371/journal.pone.0055265 23408963 PMC3567060

[40] Heberle H Meirelles GV da Silva FR Telles GP Minghim R. Interactivenn: a web-based tool for the analysis of sets through venn diagrams. BMC Bioinformatics. (2015) 16:169. 10.1186/s12859-015-0611-3 25994840 PMC4455604

[41] Li Z Fu R Huang X Wen X Zhang L. A decade of progress: bibliometric analysis of trends and hotspots in oral microbiome research (2013-2022). Front Cell Infect Microbiol. (2023) 13:1195127. 10.3389/fcimb.2023.1195127 PMC10213461 37249977

[42] Akherati M Shafaei E Salehiniya H Abbaszadeh H. Comparison of the frequency of periodontal pathogenic species of diabetics and non-diabetics and its relation to periodontitis severity, glycemic control and body mass index. Clin Exp Dent Res. (2021) 7:1080–8. 10.1002/cre2.453 34041870 PMC8638284

[43] Jung W-R Joo J-Y Lee J-Y Kim H-J. Prevalence and abundance of 9 periodontal pathogens in the saliva of periodontally healthy adults and patients undergoing supportive periodontal therapy. J Periodontal Implant Sci. (2021) 51:316–28. 10.5051/jpis.2006640332 34713993 PMC8558008

[44] Al Yahfoufi Z Hadchiti W. Prevalence of periodontal pathogens in a group of participants from the Middle East and North Africa geographic region with minimal periodontal disease. J Int Soc Prev Community Dent. (2017) 7:30. 10.4103/jispcd.JISPCD_126_17 28713765 PMC5502549

[45] Sondorová M Kučera J Kačírová J Krchová Nagyová Z Šurín Hudáková N Lipták T Prevalence of periodontal pathogens in Slovak patients with periodontitis and their possible aspect of transmission from companion animals to humans. Biology (Basel). (2022) 11:1529. 10.3390/biology11101529 36290432 PMC9598676

[46] Wang B-Y Cao A Ho M-H Wilus D Sheng S Meng H-W Identification of microbiological factors associated with periodontal health disparities. Front Cell Infect Microbiol. (2023) 13:1137067. 10.3389/fcimb.2023.1137067 36875522 PMC9978005

[47] Ebersole JL Dawson DA Emecen Huja P Pandruvada S Basu A Nguyen L Age and periodontal health—immunological view. Curr Oral Health Rep. (2018) 5:229–41. 10.1007/s40496-018-0202-2 30555774 PMC6291006

[48] Lipsky MS Su S Crespo CJ Hung M. Men and oral health: a review of sex and gender differences. Am J Mens Health. (2021) 15:15579883211016361. 10.1177/15579883211016361 33993787 PMC8127762

[49] Ioannidou E. The sex and gender intersection in chronic periodontitis. Front Public Health. (2017) 5:189. 10.3389/fpubh.2017.00189 28824898 PMC5543279

[50] Kanmaz B Lamont G Danacı G Gogeneni H Buduneli N Scott DA. Microbiological and biochemical findings in relation with clinical periodontal status in active smokers, non-smokers and passive smokers. Tob Induc Dis. (2019) 17:20. 10.18332/tid/104492 PMC6751988 31582931

[51] Aldakheel FM Alduraywish SA Jhugroo P Jhugroo C Divakar DD. Quantification of pathogenic bacteria in the subgingival oral biofilm samples collected from cigarette-smokers, individuals using electronic nicotine delivery systems and non-smokers with and without periodontitis. Arch Oral Biol. (2020) 117:104793. 10.1016/j.archoralbio.2020.104793 32544646

[52] Schulze A Mitterer F Pombo JP Schild S. Biofilms by bacterial human pathogens: clinical relevance - development, composition and regulation - therapeutical strategies. Microb Cell. (2021) 8:28–56. 10.15698/mic2021.02.741 33553418 PMC7841849

[53] Hajishengallis G Lamont RJ. Beyond the red complex and into more complexity: the polymicrobial synergy and dysbiosis (PSD) model of periodontal disease etiology. Mol Oral Microbiol. (2012) 27:409–19. 10.1111/j.2041-1014.2012.00663.x 23134607 PMC3653317

[54] Yu Y Kim H-J Song J-M Kang J Lee H Park HR Differential microbiota network in gingival tissues between periodontitis and periodontitis with diabetes. Front Cell Infect Microbiol. (2022) 12:1061125. 10.3389/fcimb.2022.1061125 36530437 PMC9755495

[55] Nobbs AH Jenkinson HF. Interkingdom networking within the oral microbiome. Microbes Infect. (2015) 17:484–92. 10.1016/j.micinf.2015.03.008 25805401 PMC4485937

[56] Grenier D. Nutritional interactions between two suspected periodontopathogens, *Treponema denticola* and *Porphyromonas gingivalis*. Infect Immun. (1992) 60:5298–301. 10.1128/iai.60.12.5298-5301.1992 1333450 PMC258310

[57] Kin LX Butler CA Slakeski N Hoffmann B Dashper SG Reynolds EC. Metabolic cooperativity between *Porphyromonas gingivalis* and Treponema denticola. J Oral Microbiol. (2020) 12:1808750. 10.1080/20002297.2020.1808750 32944158 PMC7482830

[58] de C Negrini T Carlos IZ Duque C Caiaffa KS Arthur RA. Interplay among the oral microbiome, oral cavity conditions, the host immune response, diabetes mellitus, and its associated-risk factors—an overview. Front Oral Health. (2021) 2:697428. 10.3389/froh.2021.697428 35048037 PMC8757730

[59] Hyde ER Andrade F Vaksman Z Parthasarathy K Jiang H Parthasarathy DK Metagenomic analysis of nitrate-reducing bacteria in the oral cavity: implications for nitric oxide homeostasis. PLoS One. (2014) 9:e88645. 10.1371/journal.pone.0088645 24670812 PMC3966736

[60] Barbadoro P Ponzio E Coccia E Prospero E Santarelli A Rappelli GGL Association between hypertension, oral microbiome and salivary nitric oxide: a case-control study. Nitric Oxide. (2021) 106:66–71. 10.1016/j.niox.2020.11.002 33186726

[61] LaMonte MJ Gordon JH Diaz-Moreno P Andrews CA Shimbo D Hovey KM Oral microbiome is associated with incident hypertension among postmenopausal women. J Am Heart Assoc Cardiovasc Cerebrovasc Dis. (2022) 11:e021930. 10.1161/JAHA.121.021930 PMC9075295 35234044

[62] Desvarieux M Demmer RT Jacobs DR Rundek T Boden-Albala B Sacco RL Periodontal bacteria and hypertension: the oral infections and vascular disease epidemiology study (INVEST). J Hypertens. (2010) 28:1413–21. 10.1097/HJH.0b013e328338cd36 20453665 PMC3403746

[63] Czesnikiewicz-Guzik M Nosalski R Mikolajczyk TP Vidler F Dohnal T Dembowska E Th1-type immune responses to Porphyromonas gingivalis antigens exacerbate angiotensin II-dependent hypertension and vascular dysfunction. Br J Pharmacol. (2019) 176:1922–31. 10.1111/bph.14536 30414380 PMC6534780

[64] Rahman B Al-Marzooq F Saad H Benzina D Al Kawas S. Dysbiosis of the subgingival microbiome and relation to periodontal disease in association with obesity and overweight. Nutrients. (2023) 15:826. 10.3390/nu15040826 36839184 PMC9965236

[65] Khocht A Paster B Lenoir L Irani C Fraser G. Metabolomic profiles of obesity and subgingival microbiome in periodontally healthy individuals: a cross-sectional study. J Clin Periodontol. (2023) 50:1455–66. 10.1111/jcpe.13860 37536958 PMC10749638

[66] Haffajee AD Socransky SS. Relation of body mass index, periodontitis and *Tannerella forsythia*. J Clin Periodontol. (2009) 36:89–99. 10.1111/j.1600-051X.2008.01356.x 19207883

[67] Hu X Zhang Q Zhang M Yang X Zeng T-S Zhang J-Y *Tannerella forsythia* and coating color on the tongue dorsum, and fatty food liking associate with fat accumulation and insulin resistance in adult catch-up fat. Int J Obes. (2018) 42:121–8. 10.1038/ijo.2017.191 28894293

[68] Xu J Duan X. Association between periodontitis and hyperlipidaemia: a systematic review and meta-analysis. Clin Exp Pharmacol Physiol. (2020) 47:1861–73. 10.1111/1440-1681.13372 32623762

[69] Fentoğlu Ö Tözüm Bulut M Doğan B Kırzıoğlu FY Kemer Doğan ES. Is the relationship between periodontitis and hyperlipidemia mediated by lipoprotein-associated inflammatory mediators? J Periodontal Implant Sci. (2020) 50:135–45. 10.5051/jpis.2020.50.3.135 32617179 PMC7321715

[70] Chen S Lin G You X Lei L Li Y Lin M Hyperlipidemia causes changes in inflammatory responses to periodontal pathogen challenge: implications in acute and chronic infections. Arch Oral Biol. (2014) 59:1075–84. 10.1016/j.archoralbio.2014.06.004 24992577

[71] Lei L Li H Yan F Xiao Y. Hyperlipidemia impaired innate immune response to periodontal pathogen *Porphyromonas gingivalis* in apolipoprotein E knockout mice. PLoS One. (2013) 8:e71849. 10.1371/journal.pone.0071849 23977160 PMC3745424

[72] Suh JS Kim SYJ Lee SH Kim RH Park N-H. Hyperlipidemia is necessary for the initiation and progression of atherosclerosis by severe periodontitis in mice. Mol Med Rep. (2022) 26:1–9. 10.3892/mmr.2022.12789 35795972 PMC9309540

[73] Wang Z Haslam DE Sawicki CM Rivas-Tumanyan S Hu FB Liang L Saliva, plasma, and multifluid metabolomic signatures of periodontal disease, type 2 diabetes progression, and markers of glycemia and dyslipidemia among puerto rican adults with overweight and obesity. J Am Heart Assoc. (2024) 13:e033350. 10.1161/JAHA.123.033350 39023061 PMC11964024

[74] Lönn J Ljunggren S Klarström-Engström K Demirel I Bengtsson T Karlsson H. Lipoprotein modifications by gingipains of *Porphyromonas gingivalis*. J Periodontal Res. (2018) 53:403–13. 10.1111/jre.12527 29341140 PMC5969291

[75] Wang A-Y Lin G-L Keller JJ Wang L-H. Association between antihyperlipidemic agents and the risk of chronic periodontitis in patients with hyperlipidemia: a population-based retrospective cohort study in Taiwan. J Periodontol. (2024) 95:483–93. 10.1002/JPER.23-0166 37793052

[76] de Carvalho RDP Casarin RCV de Lima PO Cogo-Müller K. Statins with potential to control periodontitis: from biological mechanisms to clinical studies. J Oral Biosci. (2021) 63:232–44. 10.1016/j.job.2021.06.002 34146687

[77] Kadhim SS Al-Windy SA Al-Nami MS Al Kuraishy HM Al Gareeb AI. Statins improve periodontal disease–induced inflammatory changes and associated lipid peroxidation in patients with dyslipidemia: two birds by one stone. J Int Oral Health. (2020) 12:66. 10.4103/jioh.jioh_194_19

[78] Ebersole JL Al-Sabbagh M Gonzalez OA Dawson DR. Aging effects on humoral immune responses in chronic periodontitis. J Clin Periodontol. (2018) 45:680–92. 10.1111/jcpe.12881 29476652 PMC5992058

[79] Matayoshi S Tojo F Suehiro Y Okuda M Takagi M Ochiai M Effects of mouthwash on periodontal pathogens and glycemic control in patients with type 2 diabetes mellitus. Sci Rep. (2024) 14:2777. 10.1038/s41598-024-53213-x 38307981 PMC10837110

[80] Bakhtiyari M Kazemian E Kabir K Hadaegh F Aghajanian S Mardi P Contribution of obesity and cardiometabolic risk factors in developing cardiovascular disease: a population-based cohort study. Sci Rep. (2022) 12:1544. 10.1038/s41598-022-05536-w 35091663 PMC8799723

[81] Liu L Xia LY Gao YJ Dong XH Gong RG Xu J. Association between obesity and periodontitis in US adults: NHANES 2011–2014. Obes Facts. (2024) 17:47–58. 10.1159/000534751 37935140 PMC10836934

